# Long-term outcomes of volume de-escalation for breast nodal irradiation

**DOI:** 10.1007/s10549-025-07652-3

**Published:** 2025-02-24

**Authors:** Riccardo Ray Colciago, Federica Ferrario, Chiara Chissotti, Giulia Rossano, Lorenzo De Sanctis, Valeria Faccenda, Denis Panizza, Sara Trivellato, Stefano Arcangeli

**Affiliations:** 1 Medicine and Surgery Department, University of Milan Bicocca, 20126 Milano, Italy; 2 Radiation Oncology Unit, Fondazione IRCCS San Gerardo Dei Tintori, Via Pergolesi 33, 20900 Monza, Italy; 3 Physics Unit, Fondazione IRCCS San Gerardo Dei Tintori, 20900 Monza, Italy

**Keywords:** Breast cancer, Regional nodal irradiation, Volume de-escalation, Long-term outcomes

## Abstract

**Introduction:**

NCCN recommendations suggest irradiating chest wall/breast only + regional node irradiation (RNI) of the undissected axillary levels for node-positive breast cancer (BC) patients. We retrospectively analyzed a cohort of node-positive BC patients who received adjuvant radiotherapy (RT) with a volume de-escalation at the level of axillary nodes.

**Material and methods:**

We conducted a retrospective analysis of node-positive BC patients treated with adjuvant RT administered following a conventional fractionation schedule using a 3D-conformal technique to the chest wall or breast and only the IV axillary level. The primary endpoint of the study was disease free survival (DFS). Secondary endpoints included loco-regional control (LRC), and Overall Survival (OS). Toxicity was documented according to the Radiation Therapy Oncology Group (RTOG) criteria.

**Results:**

A total cohort of 343 patients was analyzed. Loco-regional recurrence occurred in 100 (29.1%). The 5- and 10-year Kaplan-Meyer curves for DFS were 81.4% (95% CI: 79.3%–83.5%) and 60.9% (95% CI: 57.6%–64.5%), respectively. Multivariate Cox analysis confirmed that lymph node ratio (HR = 9.76, 95% CI: 3.12–30.53, p = 0.0001), Luminal B subtype (HR = 2.03, 95% CI: 1.26–3.29, p = 0.004), and triple-negative subtype (HR = 2.70, 95% CI: 1.22–5.99, p = 0.01) were significant predictors of poor DFS. Lymphedema in the ipsilateral arm was reported in 32 (9.3%) patients, primarily Grade 1 or 2.

**Conclusions:**

Improved patients’ selection and a broader use of systemic therapy could make de-escalation a feasible option. However, this approach should be avoided in patients with extensive nodal involvement, specific molecular subtypes, or comorbidities that prevent the use of chemotherapy.

## Introduction

The burden of breast cancer (BC) continues to grow globally [1]. From 2010 to 2019, BC incidence rose by 0.5% annually [2]. In 2020, BC became the most commonly diagnosed cancer worldwide, with an estimated 2.26 million new cases [3]. Projections suggest that by 2040, the global incidence will surpass 3 million new cases annually [4]. Additionally, the overall incidence of positive sentinel lymph nodes in BC patients is approximately 33% [5]. Lymph node involvement is associated with a higher risk of mortality and an increased likelihood of disease recurrence [6].

The treatment of nodal metastatic BC has evolved significantly over recent years. On one hand, systemic therapy has been transformed by the introduction of neoadjuvant chemotherapy, now commonly recommended for patients with nodal disease [7]. On the other hand, surgical management has shifted towards a de-escalation approach. In a landmark trial, Giuliano et al. studied 891 node-positive BC patients randomized to receive either axillary lymph node dissection (ALND) or sentinel lymph node dissection (SLND) alone. The 10-year disease-free survival (DFS) was 78.2% in the ALND group compared to 80.2% in the SLND group, with a Hazard Ratio (HR) of 0.85 [95% Confidence Interval (CI): 0.62–1.17, p = 0.32], showing no statistically significant difference.

Despite these changes, the role of radiation therapy (RT) has remained consistent. Studies have demonstrated that post-operative RT, whether following breast-conserving surgery or mastectomy for node-positive disease, reduces BC mortality [8, 9]. In the Early Breast Cancer Trialists’ Collaborative Group (EBCTCG) meta-analysis, which included 3,186 patients with nodal-positive BC who had undergone axillary dissection, RT significantly reduced locoregional recurrence, overall recurrence, and BC mortality. The benefit was even more pronounced in patients with more than four positive nodes.

Uncertainties persist regarding the appropriate radiation volumes, and clear guidelines remain undefined. National Comprehensive Cancer Network (NCCN) recommendations suggest irradiating chest wall/breast + RNI of the undissected axillary levels; however, the role of level III axillary lymph node dissection has been controversial, contributing to ambiguities about its optimal irradiation [10, 11].

Acute toxicity has decreased by employing intensity-modulated fields and contemporary approaches. More severe (exudative) skin reactions have become rare in daily practice and usually recover in a few weeks. Conversely, late reactions can be significant and lead to chronic arm lymphedema following regional irradiation, particularly in patients who undergo ALND. Further, the increasing adoption of patients’ reported outcomes (PRO) in clinical research and in daily practice can potentially strengthen this critical issue.

In this study, we aimed at investigating a possible volume de-escalation strategy to minimize treatment-related side effects without compromising tumor control. We retrospectively analyzed a cohort of node-positive breast cancer patients with long-term follow up who received adjuvant radiotherapy to the breast/chest wall and a volume de-escalation at the level of axillary nodes.

## Material and methods

### Study design

We conducted a retrospective analysis at a single institution involving patients diagnosed with invasive BC who underwent surgery with complete ALND, followed by adjuvant RT. The inclusion timeframe covered January 2009 to July 2019. Patients were excluded if they had a history of previous ipsilateral RT, underwent neoadjuvant chemotherapy, presented with bilateral nodal (N) + BC, had metastatic disease at diagnosis, or did not complete the RT course, as these factors could confound the study outcomes.

Radiation therapy was administered following a conventional fractionation schedule using 3D-conformal RT (3DCRT). Patients who received breast-targeted RT underwent also a boost of 10 Gy in 5 fractions. Target volumes included the chest wall or breast and a de-escalated approach at the axillary nodes involving only the IV level. The other axillary levels were considered as completely dissected. Indeed, per surgical practice in our institution, axillary dissection was abundant with many removed lymph nodes. Internal mammary chain was excluded from the clinical target volume (CTV). The planning target volume (PTV) includes the CTV with an isotropic margin of 7 mm. Simulation scan was a 3 mm computed tomography (CT) of the patients in a supine position. All 3D-conformal treatment plans included 6 to 15 MeV photon energies with customized, forward-planned, field-in-field approach. Additionally, image-guided RT was performed with portal imaging at the first session and every fifth session.

### Endpoints

The primary endpoint of the study was DFS—defined as the time (in months) from the end of RT to any local or systemic progression, death, or last follow-up. Secondary endpoints included loco-regional control (LRC), and Overall Survival (OS). Loco-regional control was defined as progression in the chest wall or breast and/or axillary or supraclavicular lymph nodes. Overall Survival was defined as the time between the end of RT and death. Incidence of treatment related adverse events was also measured. Toxicity was documented according to the Radiation Therapy Oncology Group (RTOG) criteria.

### Statistical analysis

Univariate and multivariate Cox proportional-hazard regressions were conducted to identify factors influencing LRC, DFS, and OS. Kaplan–Meier curves were used to estimate survival rates, with hazard ratios (HR) and 95% confidence intervals (CI) calculated for each factor. Variables with a p-value ≤ 0.10 in univariate analysis were included in multivariate models. Statistical analyses were performed using MedCalc® v23.0.2 (MedCalc Software Ltd, Ostend, Belgium), with significance defined as p ≤ 0.05.

## Results

A total cohort of 368 patients was initially considered for the study. Of these, 343 patients were analyzed, while 25 were lost to follow-up. Median follow-up was 77 months (range: 8–175). Patients and tumor characteristics are showed in Table 1

**Table 1:** Patients and Tumor Characteristics

**Age (years)**	
Median	59 (range: 27–89)
Hypertension	115 (33.5%)
Diabetes	30 (8.7%)
Dyslipidaemia	43 (12.5%)
Anticoagulants	22 (6.4%)
Smoking Habit	
Never smokers	290 (84.5%)
Active smokers	22 (6.4%)
Former smokers	27 (7.9%)
Unknown	4 (1.2%)
Median pack/year	10 (2–52)
Laterality	
Right	176 (51.3%)
Left	167 (48.7%)
Histology	
Ductal	287 (83.7%)
Lobular	52 (15.1%)
Other	4 (1.2%)
Subtype	
Luminal A	122 (35.6%)
Luminal B	126 (36.7%)
HER2 +	66 (19.2%)
TNBC	27 (7.9%)
Unknown	2 (0.6%)
Grade	
I	9 (2.6%)
II	180 (52.5%)
III	143 (41.7%)
Unknown	11 (3.2%)
LVI	214 (62.4%)
PNI	99 (28.9%)
Associated DCIS	138 (40.2%)
Margins	
Negative	297 (86.6%)
Close (< 2 mm)	13 (3.8%)
Positive	5 (1.5%)
Unknown	28 (8.1%)
Diameter	
Median	23 mm (range: 5–90)
T-Stage	
pT1	114 (33.2%)
pT2	194 (56.6%)
pT3	21 (6.1%)
pT4	6 (1.8%)
pTx	8 (2.3%)
Number of N +	
Median	7 (range: 1–28)
N-Stage	
pN1	47 (13.7%)
pN2	193 (56.3%)
pN3	103 (30.0%)
Lymph nodal Ratio	
Median	41.7% (range: 4.8–100)

*TNBC* Triple Negative Breast Cancer; *LVI* Lymphovascular Invasion; *PNI* Perineural Invasion; *DCSI* Ductal Carcinoma In Situ

### Tumor characteristics and surgical treatment

Most tumors were located on the right side (176 cases, 51.3%), with the rest on the left (167 cases, 48.7%). Breast-conserving surgery was performed in 185 cases (53.9%). The remaining 158 patients (46.1%) underwent mastectomy. The median number of lymph nodes removed was 19 (range: 6–43). Histological analysis revealed that 287 tumors (83.7%) were ductal, 52 (15.1%) were lobular, and 4 (1.2%) were classified as other types. Tumor subtypes were distributed as follows: Luminal A (122 cases, 35.6%), Luminal B (126 cases, 36.7%), HER2 positive (66 cases, 19.2%), and triple-negative breast cancer (TNBC) (27 cases, 7.9%), with 2 cases (0.6%) having unknown subtypes. The tumor grade was predominantly Grade II (180 cases, 52.5%), followed by Grade III (143 cases, 41.7%), and Grade I (9 cases, 2.6%), with grade unknown in 11 cases (3.2%).

The majority of tumors had negative surgical margins (297 case, 86.6%), 13 cases (3.8%) classified as close, 5 cases (1.5%) as positive and 28 cases (8.1%) unknown. The T-stage distribution was as follows: 114 (33.2%) T1, 194 (56.6%) T2, 21 (6.1%) T3, 6 cases (1.8%) T4, and 8 (2.3%) T unknown. The median lesion diameter was 23 mm (range: 5–90). The N-stage was predominantly N2 (193 cases, 56.3%), followed by N3 (103 cases, 30.0%), and N1 (47 cases, 13.7%). All patients (100%) underwent complete ALND. The median number of positive lymph nodes was 7 (range: 1–28), with a median lymph node ratio (number of metastatic lymph nodes/total removed lymph nodes) of 41.7% (range: 4.8–100%).

### Adjuvant therapy

Treatment characteristics are depicted in Table 2. Chemotherapy was administered to 301 patients (87.8%), while 42 patients (12.2%) did not receive chemotherapy, primarily due to their advanced age (median 79 years, range: 46–89). The most common chemotherapy regimen was a combination of taxanes and anthracyclines (267 patients, 77.8%). Anti-HER2 therapy was administered to 62 patients (18.1%). Hormonal therapy was given to 297 patients (86.6%), predominantly aromatase inhibitors (206 patients, 60.1%).

**Table 2:** Treatment Characteristics

Surgery	
Quadrantectomy	185 (53.9%)
Mastectomy	158 (46.1%)
Chemotherapy	
Yes	301 (87.8%)
*AC* + *Taxanes*	*267 (77.8%)*
*Taxanes*	*12 (3.5%)*
*FEC*	*10 (2.9%)*
*Cyclophosphamide*	*5 (1.5%)*
*AC*	*4 (1.2%)*
*Unknown*	*3 (0.9%)*
No	42 (12.2%)
Anti-HER2 therapy	62 (18.0%)
Hormonal therapy	
Yes	297 (86.6%)
*AI*	*206 (60.1%)*
*TAM*	*13 (3.8%)*
*LHRH analogous*	*78 (22.7%)*
No	45 (13.1%)
Unkonown	1 (0.3%)
Radiation therapy	
Median Dose Breast/Chest Wall	50 Gy (range: 45–60)
Median Dose Nodal Level	45 Gy
Median OTT	43 days (range: 30–72)

*AC* Anthracyclines; *FEC* 5-flourouracil + anthracyclines; *AI* Aromatase Inhibitors; *TAM* Tamoxifen; *LHRH* Luteinizing Hormone Releasing Hormone; *OTT* Overall Treatment Time

Radiation therapy was administered to the total amount of 343 patients. Of these, 185 (53.9%) received breast-targeted RT, while 158 (46.1%) were treated to the chest wall. The median dose for both the breast and chest wall was 50 Gy (range: 45–60) in 25 fractions (range: 23–30). All patients (100%) received regional node irradiation with a consistent dose of 45 Gy in 25 fractions. The median overall treatment duration was 43 days (range: 30–72).

### Toxicity

Acute toxicity was observed in 342 (99.7%) patients, with radiodermatitis reported in all of these patients. The severity of radiodermatitis was predominantly Grade 1 (244 cases, 71.1%), with 39 (11.4%) cases of Grade 2 and 59 (17.2%) cases of Grade 3. Just one patient did not develop any grade of toxicities. Acute edema occurred in 27 cases (7.8%), with the majority being Grade 1 (23 cases, 6.7%), followed by Grade 2 (3 cases, 0.9%) and Grade 3 (1 case, 0.3%). No cases of acute pneumonitis or pericarditis were recorded.

Late pulmonary fibrosis occurred in 3 (0.9%) patients, and late cardiotoxicity was observed in 6 (1.8%) patients, including valvular dysfunction, heart failure, and vascular complications. Of these, 4 were left-sided BC. Lymphedema in the ipsilateral arm was reported in 32 (9.3%) patients, primarily Grade 1 or 2. Second malignancies developed in 17 (5.0%) patients, with lung cancer and hematologic malignancies being the most common.

### Clinical outcomes

**Fig. 1.**
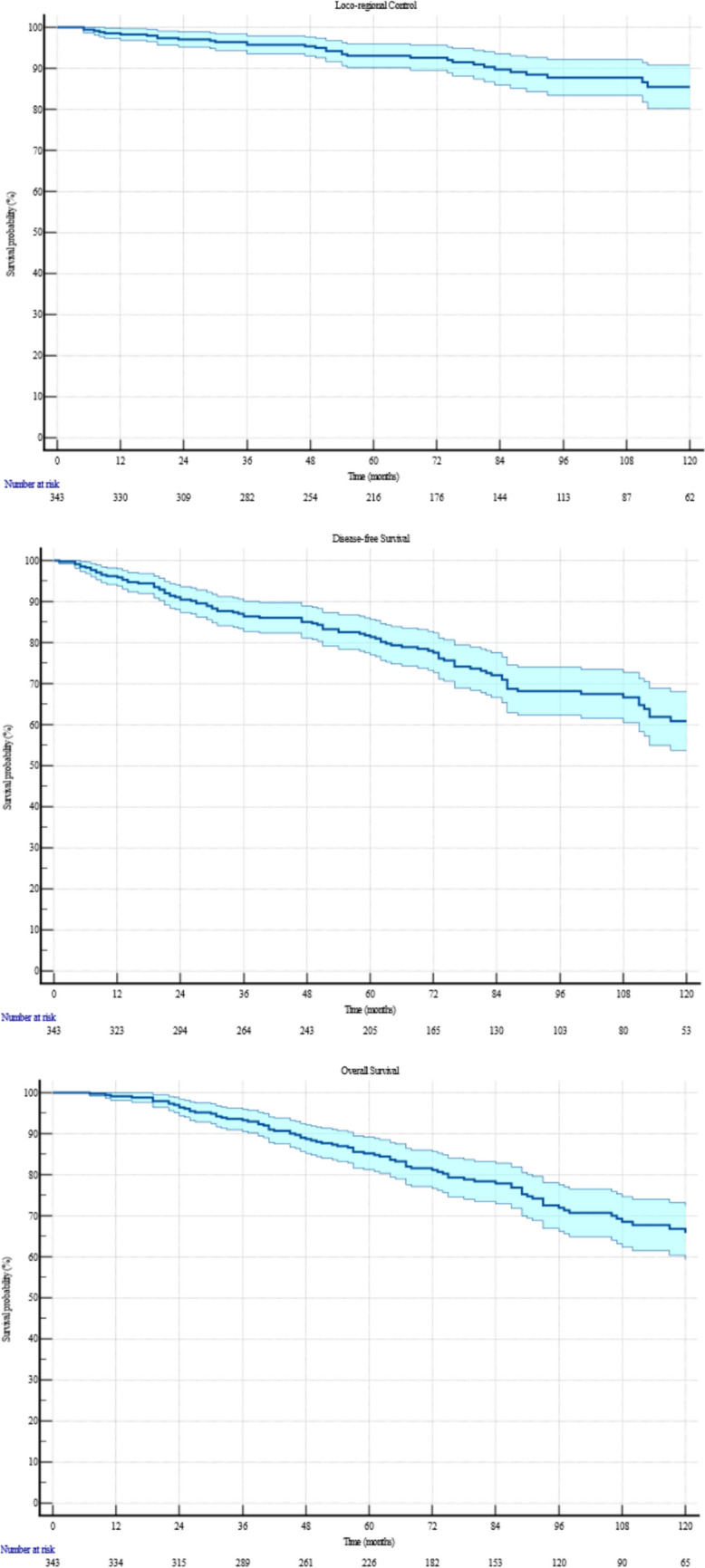
10-years Kaplan-Meyer for LRC, DFS and OS

Loco-regional recurrence occurred in 39 (11.4%) patients, with a median control duration of 74 months (range: 5–173). Specifically, 8 (2.3%) patients had local recurrences, 20 (5.8%) had regional recurrences, and 11 (3.2%) experienced both. The 5- and 10-year Kaplan–Meier estimates for loco-regional control were 93.0% (95% CI: 91.5%–94.5%) and 85.4% (95% CI: 81.7%–88.1%), respectively. Figure 1 depicts the Kaplan-Meyer for LRC, DFS and OS. Univariate Cox analysis identified a strong correlation between the Luminal B subtype and an increased risk of loco-regional recurrence (HR = 3.01, 95% CI: 1.34–6.76; p = 0.0078). This correlation was further confirmed in multivariate Cox analysis, where being Luminal B remained a significant factor (HR = 2.84, 95% CI: 1.25–6.47; p = 0.0127). Table 3 synthetize all correlation analysis.

**Table 3:** Factors Related to Survival Outcomes

		*Variable*	*HR*	*95% CI*	*p-value*
Loco-Regional Control 10-years: 85.4%	*Univariate*				
		*Luminal B*	3.00	1.33–6.75	***0.007***
		*TNBC*	3.22	0.95–10.74	*0.06*
		*Number of N* +	1.03	0.99–1.07	*0.06*
		*Lymph nodal Ratio*	2.98	0.94–9.47	*0.06*
		*Pack/year*	1.02	0.99–1.05	0.07
		*OTT*	1.03	0.99–1.07	0.08
		*TAM*	2.85	0.85–9.56	0.08
	*Multivariate*				
		*Luminal B*	2.84	1.24–6.46	***0.01***
		*OTT*	1.04	0.99–1.08	*0.06*
Disease Free Survival 10-years: 60.9%	*Univariate*				
		*Lymph nodal Ratio*	6.00	2.92–12.31	** < *0.001***
		*Number of N* +	1.04	1.01–1.06	***0.001***
		*pN3*	3.13	1.53–6.42	***0.001***
		*Luminal B*	1.81	1.14–2.87	***0.01***
		*TNBC*	2.34	1.13–4.82	***0.02***
		*Lobular*	1.51	0.94–2.43	0.08
	*Multivariate*				
		*Lymph nodal Ratio*	9.75	3.11–30.5	** < *0.001***
		*Luminal B*	2.03	1.25–3.28	***0.003***
		*TNBC*	2.70	1.22–5.98	***0.01***
Overall Survival 10-years: 65.8%	*Univariate*				
		*Age*	1.03	1.02–1.05	** < *0.0001***
		*Anticoagulants*	4.42	2.52–7.74	** < *0.0001***
		*Chemotherapy*	0.27	0.17–0.44	** < *0.0001***
		*Number of N* +	1.04	1.02–1.07	***0.0001***
		*Lymph nodal Ratio*	4.03	1.95–8.34	***0.0002***
		*pN3*	5.51	2.16–14.01	***0.0003***
		*Diabetes*	2.55	1.51–4.33	***0.0005***
		*Hypertension*	2.03	1.35–3.04	***0.0006***
		*pN2*	3.38	1.33–8.54	***0.01***
		*TNBC*	2.48	1.17–5.27	***0.01***
		*HT*	0.56	0.33–0.95	***0.04***
	*Multivariate*				
		*Number of N* +	1.05	1.02–1.07	***0.0001***
		*Chemotherapy*	0.42	0.22–0.79	***0.01***
		*Anticoagulants*	2.56	1.22–5.36	***0.01***
		*Luminal B*	1.77	1.07–2.94	***0.02***

In [bold] are the statistically significant p-values

*TNBC* Triple Negative Breast Cancer; *OTT* Overall Treatment Time; *TAM* Tamoxifen; *HT* Hormonal Therapy; *pN* Pathological Nodal Stage

Disease recurrence occurred in 100 (29.2%) patients, with a median onset time of 70 months (range: 1–173). The 5- and 10-year actuarial rates for DFS were 81.4% (95% CI: 79.3%–83.5%) and 60.9% (95% CI: 57.6%–64.5%), respectively. Univariate Cox analysis identified significant associations between DFS and the absolute number of positive lymph nodes (HR = 1.04, 95% CI: 1.02–1.07, p = 0.0005) and lymph node ratio (HR = 6.00, 95% CI: 2.92–12.31, p < 0.0001). Patients with 1–3 positive lymph nodes had a median DFS of 70 months, with 5- and 10-year rates of 84.7% (95% CI: 79.0%–91.4%) and 74.4% (95% CI: 65.6%–83.2%), respectively. Those with more than 4 positive nodes also had a median DFS of 70 months, but their 5- and 10-year rates were lower, at 80.9% (95% CI: 78.6%–83.2%) and 58.9% (95% CI: 55.0%–62.8%), respectively. Additionally, T2-stage tumors (HR = 1.63, 95% CI: 1.02–2.58; p = 0.04), N3 classification (HR = 3.14, 95% CI: 1.53–6.42; p = 0.002), Luminal B subtype (HR = 1.81, 95% CI: 1.14–2.87; p = 0.01), and TNBC subtype (HR = 2.34, 95% CI: 1.14–4.83; p = 0.02) were also associated with worse DFS outcomes. Multivariate Cox analysis confirmed that lymph node ratio (HR = 9.76, 95% CI: 3.12–30.53, p = 0.0001), Luminal B subtype (HR = 2.03, 95% CI: 1.26–3.29, p = 0.004), and TNBC subtype (HR = 2.70, 95% CI: 1.22–5.99, p = 0.01) were significant predictors of poor DFS.

A total of 98 (28.6%) deaths were recorded, with a median OS of 76 months (range: 6–173). The 5- and 10-year actuarial rates for OS were 85.2% (95% CI: 83.2%–87.2%) and 65.8% (95% CI: 62.4%–69.2%), respectively. In the Cox univariate analysis, significant associations with OS were observed across multiple factors: older age (HR = 1.04, 95% CI: 1.02–1.06; p < 0.0001) and anticoagulants use (HR = 4.42, 95% CI: 2.52–7.75; p < 0.0001) showed strong correlations as risk factors. Hypertension (HR = 2.03, 95% CI: 1.36–3.05; p = 0.0006), diabetes (HR = 2.56, 95% CI: 1.51–4.33; p = 0.0005), dyslipidemia (HR = 2.26, 95% CI: 1.37–3.71; p = 0.0013), and the number of positive lymph nodes (HR = 1.05, 95% CI: 1.02–1.07; p = 0.0001) were also significantly associated with worse OS outcomes. For patients with 1 to 3 positive lymph nodes, the 5- and 10-year actuarial OS rates were 92.3% (95% CI: 88.0%–96.6%) and 76.5% (95% CI: 67.4%–85.6%), respectively, while for those with more than 4 positive nodes, these rates were 84.1% (95% CI: 81.9%–86.3%) and 64.4% (95% CI: 60.8%–68.0%), respectively. Lymph node ratio (HR = 4.04, 95% CI: 1.95–8.35; p = 0.0002), nodal stages N2 (HR = 3.38, 95% CI: 1.34–8.55; p = 0.01) and N3 (HR = 5.51, 95% CI: 2.17–14.01; p = 0.0003), as well as the Luminal B subtype (HR = 1.64, 95% CI: 1.02–2.63; p = 0.042) and TNBC subtype (HR = 2.49, 95% CI: 1.17–5.28; p = 0.017), were significant risk factors for OS. Conversely, undergoing hormonal therapy was associated with a protective effect (HR = 0.57, 95% CI: 0.34–0.95; p = 0.033), and chemotherapy demonstrated a strong protective association (HR = 0.28, 95% CI: 0.17–0.45; p < 0.0001). In the Cox multivariate analysis, anticoagulants use (HR = 2.56, 95% CI: 1.22–5.36; p = 0.013) and higher number of positive lymph nodes (HR = 1.05, 95% CI: 1.03–1.08; p = 0.0001) remained significant predictors of poor OS. The Luminal B subtype (HR = 1.78, 95% CI: 1.07–2.95; p = 0.025) continued to show a significant correlation with reduced OS, while chemotherapy maintained its protective role (HR = 0.42, 95% CI: 0.22–0.80; p = 0.008).

## Discussion

Lymph node involvement is associated with a higher risk of mortality and an increased likelihood of disease recurrence [6]. Studies have shown that postoperative RT, whether after breast-conserving surgery or mastectomy for node-positive disease, significantly reduces BC mortality [8, 9]. The EBCTCG published a meta-analysis of 22 older trials involving 3,186 patients with node-positive BC who underwent axillary dissection. RT was found to significantly reduce locoregional recurrence, DFS, BC specific and overall mortality [9]. Additionally, in a more recent meta-analysis, the EBCTCG demonstrated that RT to regional nodes offers greater benefits to patients with a higher burden of disease. They analyzed the outcomes of 14,324 patients from 16 trials, finding that the estimated absolute reductions in 15-year BC mortality were 1.6%, 2.7%, and 4.5% for patients with no, one to three, and four or more positive axillary nodes, respectively [12].

However, uncertainties persist regarding the appropriate radiation volumes, and clear guidelines remain undefined. This is likely due to the fact that trials conducted in the 1990s and 2000s were insufficient to determine whether RT is beneficial based on factors such as tumor location, nodal involvement, or the use of systemic therapy [13–15]. Consequently, clinical guidelines have shown significant variability [16, 17]. Additionally, the surgical landscape has evolved following several randomized trials that demonstrated the potential to omit complete axillary dissection in patients with low-burden nodal disease [18–20]. Taken together these four trials randomized 4,246 breast cancer patients with one to two positive axillary lymph nodes to either undergo ALND or SLND alone. The results indicated that SLND alone could provide comparable disease control to ALND in this population. However, limited data remains regarding adjuvant treatments, particularly RT indications, doses, and volumes, which has further contributed to the existing uncertainty encountered by radiation oncologists in their clinical practice of [21].

The NCCN guidelines recommend irradiating chest wall/breast + RNI of the undissected axillary levels; however, the role of level III axillary lymph node dissection remains controversial, adding further uncertainty about its optimal irradiation [10, 11]. In this study, we aimed to explore a possible volume de-escalation approach for axillary node irradiation. Treatment optimization is a central focus for radiation oncologists, with the goal of improving patient selection and identifying opportunities for volume de-escalation without compromising tumor control [22]. Our objective was to achieve effective disease control while minimizing treatment-related toxicities by irradiating only the level IV axillary nodes, considering level III sufficiently treated with surgical dissection.

In total, we observed 39 (11.3%) loco-regional recurrences only, 100 (29.1%) disease relapses at any site (loco-regional and/or distant), and 98 (28.5%) deaths. The 10-year actuarial LRC rates we reported are comparable to those in the EBCTCG meta-analysis [9]. In their analysis, the authors included 22 trials that began before 2000, comparing adjuvant RT with no RT after mastectomy for BC. Among the 8,135 patients, 3,131 had positive pathological nodal status and underwent axillary dissection. For these patients, the 10-year LRC was 91.9% in the RT group compared to 74.0% in the no-RT group. Our results fall in between, with a 10-year LRC Kaplan–Meier estimate of 85.4%. However, our dataset includes a higher proportion of patients with more than four positive lymph nodes (86.3 vs 56.5%). In the sub-analysis of the 1,772 pN4 + patients from the EBCTCG meta-analysis, the 10-year actuarial LRC rate was comparable to our results with an actuarial rate of 87.0%. The low incidence of disease recurrence observed, despite the reduced radiation volume, could largely be attributed to the introduction of new chemotherapy agents, which have been shown to improve disease control and survival compared to the systemic therapies analyzed in the EBCTCG meta-analysis [23]. However, in the absence of these new agents, it is likely that volume de-escalation of RNI would have led to worse disease control, particularly in high-risk patients. Additionally, loco-regional control may also be influenced by dose diffusion from level IV axillary nodes to adjacent levels (primarily level III) due to the 3D irradiation technique [24].

In terms of DFS, our data are disappointing. Compared to the rates from the latest EBCTCG meta-analysis, our 5-year and 10-year DFS rates are 81.4% and 60.9%, respectively, versus 82.2% and 73.8% [12]. But this discrepancy widens when considering only the eight newer trials, which report 5-year and 10-year DFS rates of 85.2% and 77.1%. While the 5-year DFS rates are comparable, the 10-year outcomes are poorer. Although this difference can be partly attributed to the higher rates of loco-regional failure in our study (14.6% vs 5.1% in the meta-analysis), a worse DFS may also be due to an inferior control of distant metastases.

Albeit some studies indicate that loco-regional RT can improve distant metastasis control, the underlying rationale remains unclear [25]. The primary reason for a reduced DFS in our cohort might be attributed to patients’ selection and differences in systemic treatments. We found that the number of positive lymph nodes, lymph node ratio, and molecular subtype were associated with worse DFS outcomes. Specifically, patients with pN1-3 disease had 5- and 10-year actuarial DFS rates of 92.3% and 76.5%, respectively, while those with pN4 + disease had rates of 84.1% and 64.4%. Luminal B and triple-negative tumors accounted for 44.9% of cases in our cohort, and both subtypes are known to be associated with poorer prognosis [26, 27]. Additionally, the median lymph node ratio in our study was at 41.7%. Vinh-Hung et al. [28] analyzed lymph node ratios in 1,829 node-positive BC patients and classified them into three groups: Low-risk (≤ 20%), intermediate-risk (> 20% and ≤ 65%), and high-risk (> 65%). In the intermediate-risk group, where our median value falls, the 10-year DFS rate was 63%.

Ultimately, the role of RT in OS remains a topic of debate. In the early 2000s, Rutqvist et al. [29] conducted a comprehensive review of 29 randomized trials, 6 meta-analyses, and 5 retrospective studies, including a total of 285,982 patients. They found that while BC-specific survival improved with postmastectomy RT, non-BC-specific survival was reduced. In 2005, Clarke et al. [30] analyzed data from 42000 patients across 78 randomized studies and found a significant increase in non-BC mortality among irradiated women, primarily due to heart disease and lung cancer. Similarly, the 2011 EBCTCG meta-analysis [8] concluded that RT did not significantly improve mortality reduction. However, recent findings from the same study group indicate a significant improvement in OS for BC patients who received adjuvant RT, but this benefit was evident only when considering the 8 newer studies (15-year gain 3.0%, HR 0.90; p = 0.002) [12]. This improvement is largely attributed to advances in technology that have reduced heart exposure to radiation. In these newer studies, the mortality rate without recurrence was similar in both groups (15-year gain 0.6%, HR 0.97; p = 0.63). In our cohort, the 10-year OS rate was 65.8%, comparable to the 64.3% reported in the 8 older trials of the latest EBCTCG meta-analysis [12]. This survival rate is likely influenced by patient selection and the administration of chemotherapy. Our analysis identified adjuvant chemotherapy as a significant protective factor (HR 0.4; p = 0.007) as supported by the literature [31]. However, 12.5% of our patients did not receive systemic chemotherapy.

Regarding toxicities, the most significant issue in the context of nodal irradiation is late lymphedema. In our series, lymphedema incidence was low, with only 32 cases (9.3%). The AMAROS trial, conducted by Donker et al. [32], randomized 4,806 BC patients to either ALND or axillary RT. At 5 years, lymphedema was reported in 23% of the ALND group compared to 11% in the RT group. Our results show a lower incidence than the ALND group, which suggests potential limitations in the study. Indeed, the retrospective nature of our study may introduce biases in event detection and reporting. Additionally, while the median follow-up is considerable, it does not reach 15 years, which may lead to an underestimation of longer-term outcomes. Breast cancer is known for late recurrences, necessitating a longer follow-up period [33, 34]. Despite these limitations, our findings provide valuable real-world insights into long-term volume de-escalation in patients with nodal positive BC.

## Conclusions

The de-escalation of irradiated regional volumes in node-positive breast cancer patients who have undergone surgery and complete ALND has yielded controversial results. For those with extensive nodal involvement, specific molecular subtypes, or comorbidities that preclude chemotherapy, this approach should be strongly discouraged.

Future trials are needed to determine whether improved patient selection—incorporating biological and molecular assays—along with the broader use of systemic therapy, will guide decision-making on which patients can safely undergo volume de-intensification.

## Data Availability

The datasets used and/or analysed during the current study are available from the corresponding author on reasonable request.
